# The safety and efficacy of high-speed train transport for critical children: a retrospective propensity score matching cohort study

**DOI:** 10.1038/s41598-021-98944-3

**Published:** 2021-09-29

**Authors:** Zhe Zhao, Yingyue Liu, Baowang Yang, Huiling Zhang, Xiaohong Liu, Yanjuan Zhu, Xiaoyang Hong, Zhichun Feng

**Affiliations:** 1 The Second School of Clinical Medicine, Southern Medical University, Guangzhou, China; 2 Pediatric Intensive Care Unit, Senior Department of Pediatrics, The Seventh Medical Center of Chinese PLA General Hospital, Beijing, China

**Keywords:** Paediatric research, Health care economics

## Abstract

It is widely acknowledged that efficiency of pediatric critical care transport plays a vital role in treatment of critically-ill children. In developing countries, most critically-ill children were transported by ambulance, and a few by air, such as a helicopter or fixed airplane. High-speed train (HST) transport may be a potential choice for critically-ill children to a tertiary medical center for further therapy. This is a single-center, retrospective cohort study from June 01, 2016 to June 30, 2019. All the patients transported to the Pediatric Intensive Care Unit (PICU) of PLA general hospital were divided into two groups, HST group and ambulance group. The propensity score matching method was performed for the comparison between the two groups. Finally, a 2:1 patient matching was performed using the nearest-neighbor matching method without replacement. The primary outcome was hospital mortality. Secondary outcomes included duration of transport, transport cost, hospital stay, and hospitalization cost. A total of 509 critically-ill children were transported and admitted. Of them, 40 patients were transported by HST, and 469 by ambulance. The hospital mortality showed no difference between the two groups (*p* > 0.05). The transport distance in the HST group was longer than that in the ambulance group (1894.5 ± 907.09 vs*.* 902.66 ± 735.74, *p* < 0.001). However, compared to the HST group, the duration of transport time by ambulance was significantly longer (*p* < 0.001). No difference in vital signs, blood gas analysis, and critical illness score between groups at admission was noted (*p* > 0.05). There was no death during the transport. There was no difference between groups regarding the transport cost, hospital stays, and hospitalization cost (*p* > 0.05). High-quality tertiary medical centers are usually located in megacities. HST transport network for critically-ill children could be established to cover most regions of the country. Without increasing financial burden, HST medical transport can be a potentially promising option to improve the outcomes of critically-ill children in developing countries with developed HST network.

Clinical Trial Registration: This study was registered at http://www.chictr.org.cn/index.aspx (chiCTR.gov; Identifier: ChiCTR2000032306).

The vital role of pediatric critical care transport has been widely acknowledged in the past 30 years with evidence showing that a pediatric transport team can improve the outcomes of critically-ill children^1,2^. Currently, most critically-ill children are transported by ambulance and a few by air, such as helicopters or fixed airplanes. Lack of air transport service is a big challenge to the health system in some remote regions. The advantages of ambulance transport include low cost, point to point, and no need for a parking apron like for a helicopter. However, the shortcoming is also apparent, such as longer duration, due to road and weather conditions^3^. Air medical transport is quite common in developed countries, which is faster and free from traffic jams. In the case of transport of over 500 km, air medical transport is the best choice^4^. However, the cost of air medical transport is unaffordable for many families in developing countries, like China, as it is not covered by the National Insurance System. It is urgent to develop an innovative way to transport critically-ill children for over 500 km. High-speed trains (HST) has been developing rapidly in China in recent years. As of in 2019, the HST system has covered almost all parts of China except some remote areas in the west, with a total railway distance of over 35,000 km^5^. The speed of the HST in China could reach up to 350 km per hour, and the HST network has connected most of the regional medical centers in China, making it a promising innovative modality for long-distance transport^6^. Although this innovative transport modality has been carried out in China for a few years, its safety and efficacy have not been well defined. This study aimed to clarify whether transportation by HST has superior outcomes compared to ambulance transport.

## Methods

### Ethical approvals

The study was approved by the Ethics Committee of the People’s Liberation Army general hospital (No.2016-039) (Beijing, China), and registered in ChiCTR (ChiCTR2000032306). Written informed consent was obtained from the parents. The methodology in this study was in accordance with the relevant guidelines and regulations.

### Study design

This is a single-center, retrospective cohort study, conducted from June 01, 2016 to June 30, 2019. A total of 509 pediatric patients under 14 years old, who were transported to a tertiary medical center, were recruited in this study. All the children were confirmed with an indication of transport^7^. Of them, 40 children were transported by HST, and 469 children by ambulance.

### HST carriage for medical transport

**Figure 1:**
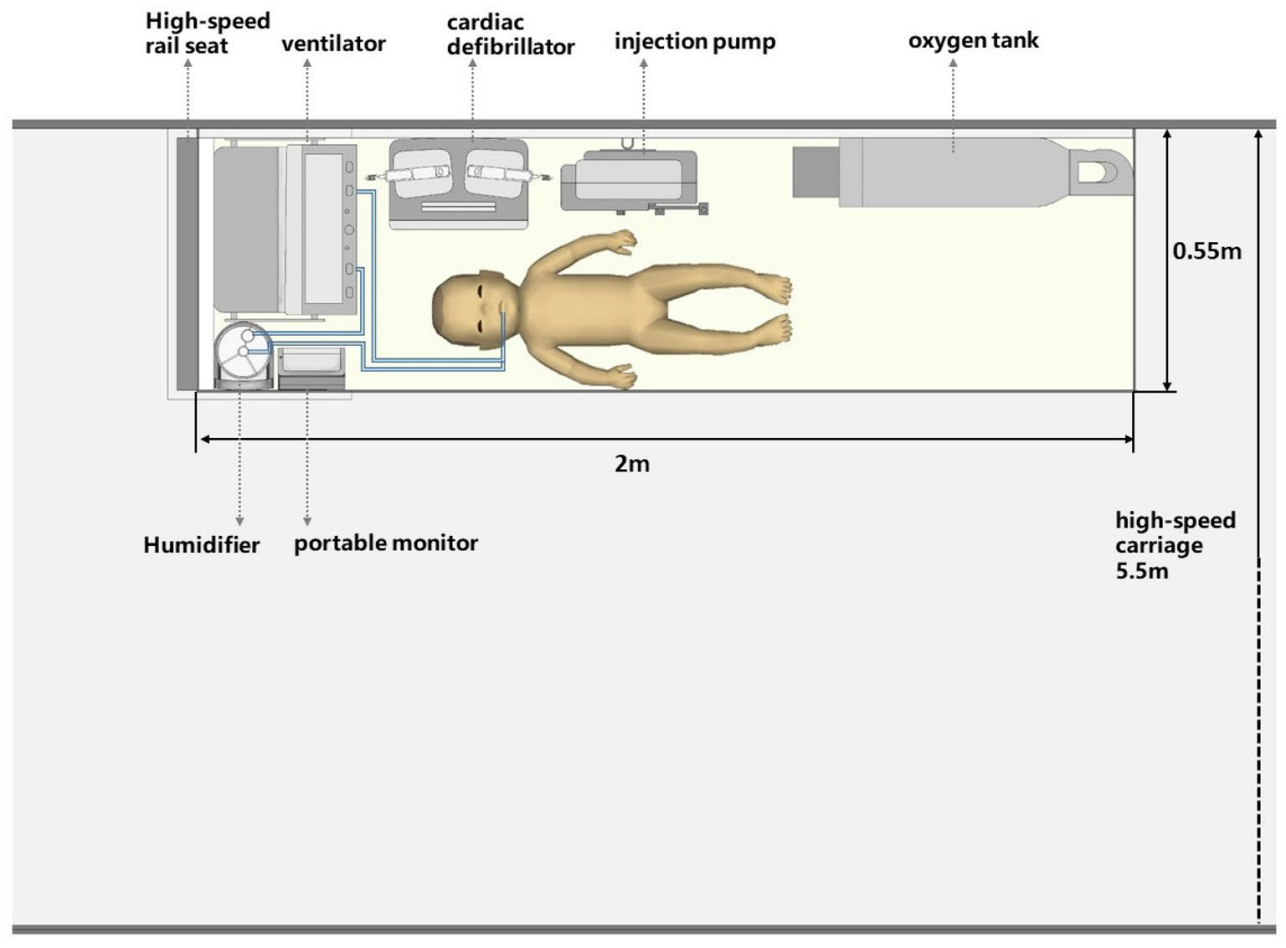
Floor plan of HST carriage during critically-ill children transportation. The transport stretcher, 0.55 m wide and 2 m long, is placed behind the business class seat. Equipment placement are shown in the figure, including a ventilator, monitor, infusion pump, oxygen pipe, etc.

**Figure 2:**
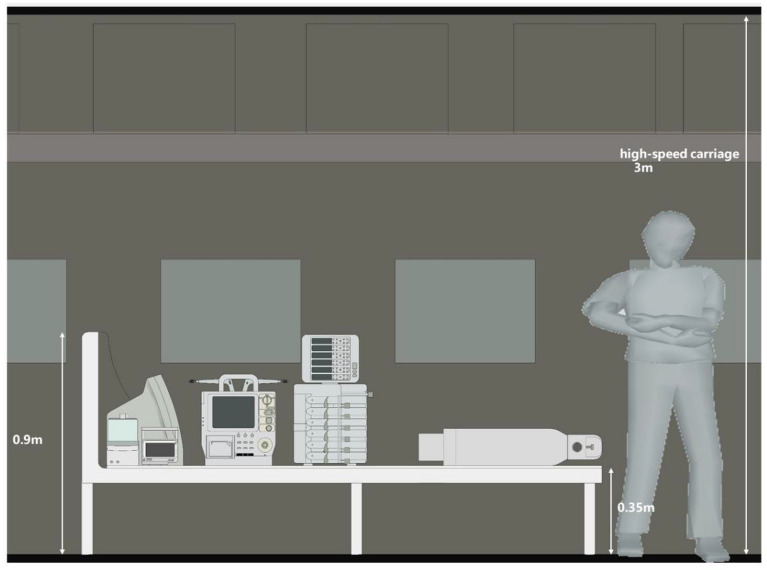
Side view of HST carriage during critically-ill children transportation. The height of the transfer stretcher is 0.9 m, and the height of the carriage is 3 m.

A modification of the HST carriage was necessary to fit the requirement of critically-ill children's transport. Business-class of HST has enough space to accommodate a medical stretcher and other equipment. Under special conditions, the business-class seat could be regulated to make room for a stretcher for patients. The necessary equipment during transport includes a ventilator, infusion pumps, cardiopulmonary monitor, blood gas analyzer, vacuum extractor, intubation supplies, oxygen cylinder or oxygen generator et al. (Figs. 1 and 2).

### Clinical management

Our HST transport program was set up in 2016. All the medical care was provided by our pediatric/neonatal critical care transport team (CCTT). In this program, CCTT was composed of a neonatal and pediatric intensivist and a registered nurse. A checklist of HST transport was reviewed before leaving. During transport, routine management mainly includes intravenous administration of medication, and respiratory supports, including oxygen supply and mechanical ventilation. The choice of respiratory support during transport was dependent upon the comprehensive clinical evaluation. Prostaglandin E1 is considered for some children with patent ductus arteriosus dependent congenital heart disease.

The vital signs, including heart rate, respiratory rate, blood pressure, and peripheral oxygen saturation, were closely monitored during transport. End-tidal carbon dioxide (ETCO2) was placed on the children who needed invasive mechanical ventilation. The vital signs and relevant clinical conditions were regularly recorded in the transportation sheet, and blood gas analysis was obtained immediately at admission to the referral hospital. The critical illness score was assessed and recorded after admission.

### Outcomes

The primary outcome was hospital mortality. Secondary outcomes included duration of transport, transport cost, hospital stay and hospitalization cost.

### Data collection

All data encompassing demographic data, vital signs, medication, procedure, diagnosis, and other relevant information were extracted from the transport record database. Blood gas analysis was retrospectively read from the hospital information systems.

### Statistical analysis

To minimize the effect of confounding factors and potential bias between the ambulance and HST groups, the propensity score was calculated using logistic regression, and a 2:1 patient matching was performed using the nearest-neighbor matching method without replacement. Variables in the matching model were gender, age, body weight, diagnosis, respiratory supports, the pediatric risk of mortality III (PRISMIII), and Score for neonatal acute physiology II (SNAPII). The median of continuous and ordinal variables and the exact conditional maximum likelihood estimation of binary variables were calculated. The characteristics and outcomes of each pair were compared based on the exact sign tests and 95% confidence intervals for the median and estimation.

## Results

### Working flow and patients’ selection

**Figure 3:**
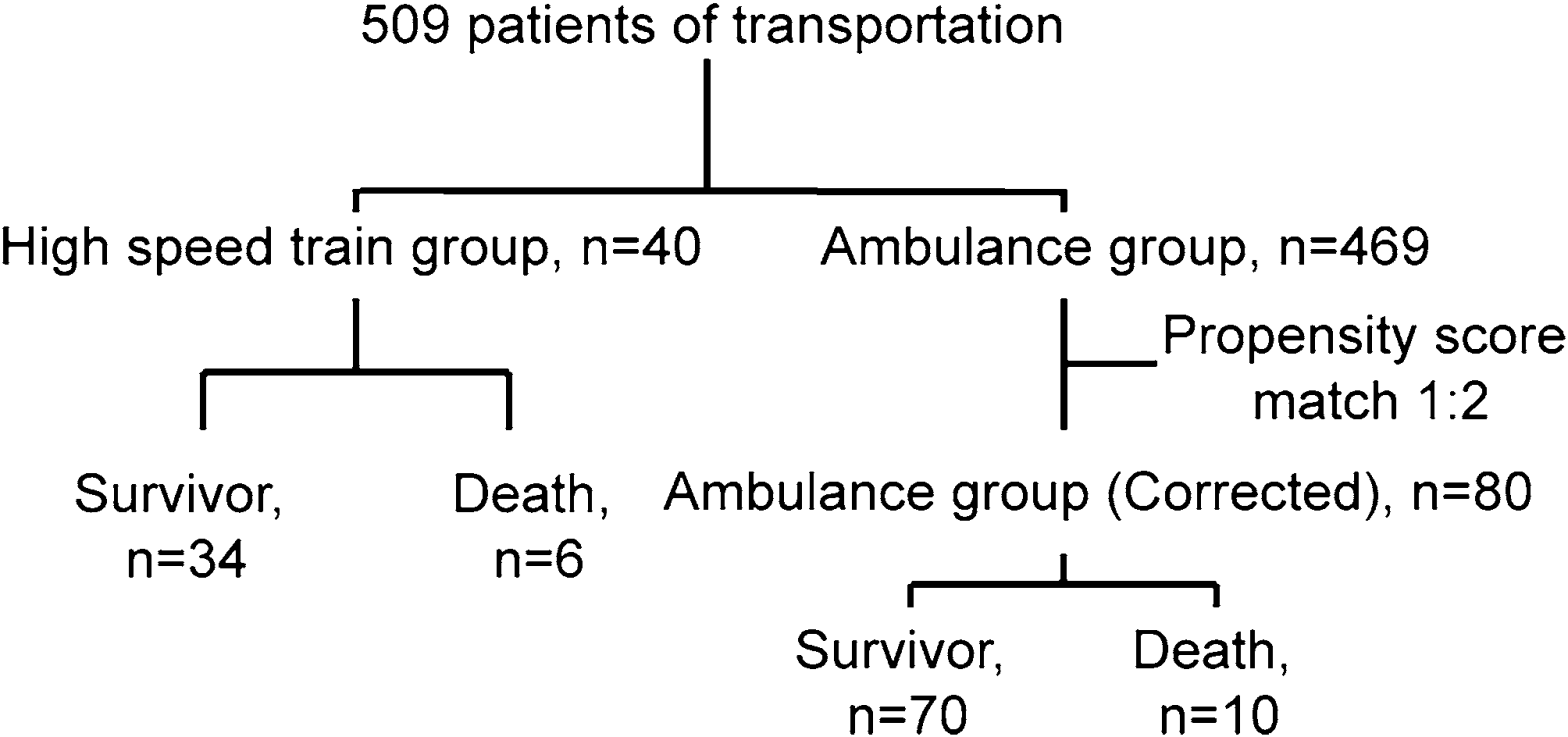
Workflow for the matching of subjects in 509 children in this study.

A total of 509 children were transported to PICU between June 01, 2016 and June 30, 2019. Among them, 40 cases were transported by HST, and 469 by ambulance. Propensity score matching (PSM) of 1:2 was performed, and 40 cases in the HST group and 80 cases in the ambulance group were eligible for the analysis in this study. Among them, six and ten children were dead in the HST group and the ambulance group respectively (Fig. 3).

### Basic information of HST and ambulance group after PSM

After matching, covariates showed a fine balance for all matched variables. The demographic characteristics, including gender, age and body weight did not show any difference between the HST and ambulance group (*p* > 0.05), nor did the diagnosis and respiratory supports during transport (*p* > 0.05). The critical illness scores, including pediatric risk of mortality III (PRISMIII) score and Score for neonatal acute physiology II (SNAPII), also showed no difference between the two groups (*p* > 0.05). However, as the distance between the referring hospital and the receiving hospital differed greatly (1894.5 ± 907.09 vs*.* 902.66 ± 735.74, *p* < 0.001), the transport distance between the two groups was not included for the calculation of propensity score.

### Vital signs, blood gas analysis and critical illness score at admission

Vital signs, including heart rate, respiratory rate, systolic pressure, diastolic pressure, and rectal temperature showed no difference between HST and the ambulance group (*p* > 0.05). The vital parameters of blood gas analysis, including pH, PaO_2_, PaCO_2,_ and BE, also showed no difference between groups at admission (*p* > 0.05). The level of lactate in the ambulance group was higher than that in the HST group (2.73 ± 2.76 mmol/L vs.1.66 ± 1.21 mmol/L, *p* = 0.006). There was no difference in the critical illness score between groups at admission (*p* > 0.05) (Table 1).

**Table 1:** Comparison of vital signs and blood gas analysis between two groups at admission.

	Ambulance group	HST group	t	*P*
	(n = 80)	(n = 40)		
**Vital signs at admission**				
HR	139.49 ± 19.24	138.20 ± 17.49	0.356	0.723
RR	39.71 ± 10.68	39.13 ± 9.09	0.298	0.766
Systolic pressure(mmHg)	78.43 ± 15.85	78.92 ± 10.07	− 0.172	0.864
missing(n)	1	3		
Diastolic pressure(mmHg)	44.42 ± 10.67	43.62 ± 5.83	0.520	0.604
missing(n)	2	3		
Rectal temperature	36.80 ± 0.61	36.67 ± 0.40	1.209	0.229
**Blood gas analysis at admission**				
pH	7.40 ± 0.12	7.42 ± 0.08	− 0.820	0.414
missing (n)	4	0		
PaO_2_	110.77 ± 88.47	104.18 ± 53.50	0.431	0.668
missing (n)	4	0		
PaCO_2_	44.72 ± 15.80	43.61 ± 12.63	0.385	0.701
missing (n)	4	0		
Lactate	2.55 ± 2.28	1.66 ± 1.21	2.667	0.009*
missing (n)	11	0		
BE	1.87 ± 6.00	2.30 ± 4.44	− 0.394	0.694
missing (n)	9	0		
**Critical illness score**				
PRISMIII	6.24 ± 5.80	5.39 ± 4.82	0.603	0.548
SNAPII	8.29 ± 8.36	7.94 ± 9.07	0.138	0.891

HR: heart rate; RR: respiratory rate; BE: base excess; PRISMIII, the pediatric risk of mortality III, SNAPII, Score for neonatal acute physiology II, SNAPII; *p < 0.05.

### Outcomes

There was no death during transport in both groups. The hospital mortality rate was 12.5% in the ambulance group, compared to 15.0% in the HST group without significant difference (*p* = 0.704). There was no difference in transport cost, hospital stays and hospitalization cost between HST and ambulance groups (*p* > 0.05). Compared to the HST group, the duration of transport by ambulance was significantly longer than that of HST (*p* < 0.001).

## Discussion

To our knowledge, this is the first clinical study on HST transport for critically-ill pediatric patients. To our knowledge, most critically-ill pediatric patients were transported by ambulance in developing countries, like China. Due to the limitation of ambulance transport and the high cost of air medical transport, long-distance transport of critically-ill children is still a challenge in developing countries. In this study, our findings suggest that critically-ill children can be transported by HST safely and efficiently, without increasing mortality rate and cost. HST can play an essential role on critically-ill children transport in developing countries and countries with the HST network.

**Figure 4:**
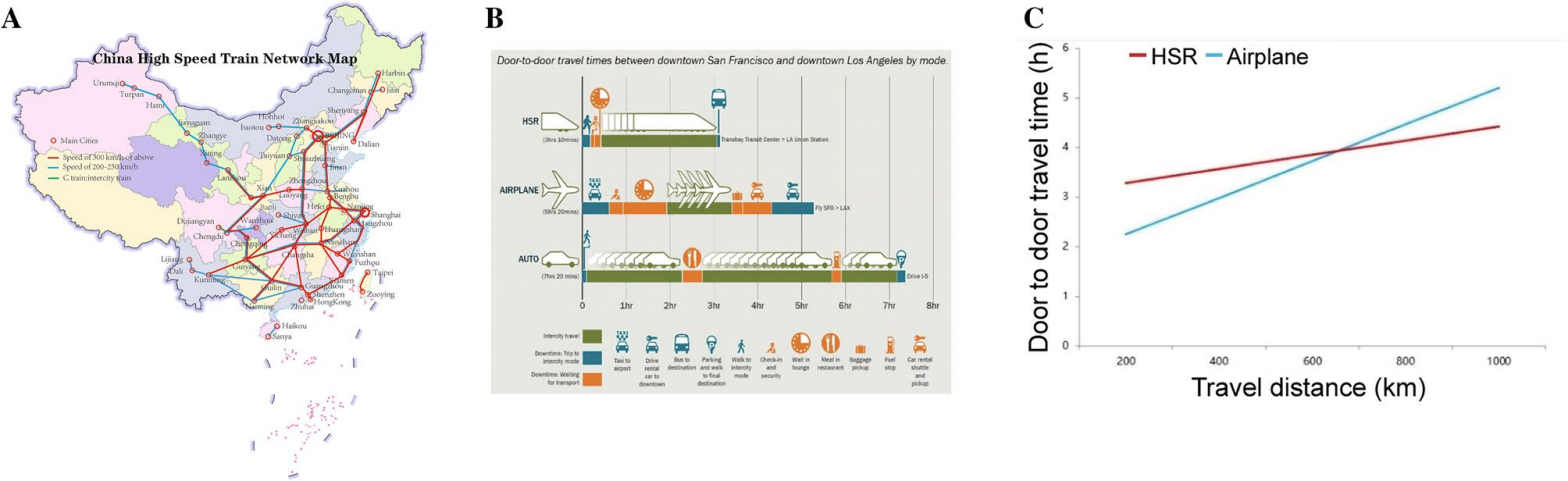
(**A)** China HST Network Map (updated on December 2019). The map was created with Adobe Illustrator CS5 (US), https://www.adobe.com/cn/products/illustrator.html. (**B**) Door-to-door travel time between San Francisco and Los Angeles by different transports. (**C**) Door-to-door travel time by different distances between high-speed train and airplane.

A total of 509 children were transported to our PICU from June 01, 2016 to June 30, 2019, but only 40 cases were transported by the HST. HST transport was not suitable for all children. Firstly, the distance between the two hospitals would limit the use of HST. In regards to patients living close to Beijing, the use of HST would spend much more time than transport by ambulance as the transport between the hospital and the HST station need extra time. As shown in Fig. 4B, transport by HST follows the “referring hospital-ambulance-HST-ambulance-receiving hospital” mode, which needs good coordination and more transfer time between different nodes. Secondly, patients receiving treatment have to be in a city with an HST station. The hospital requesting the transportation service should be close to an HST station so that the patients could be transported timely by ambulance to catch the targeted HST immediately.

It is also convenient for the children to be transported to a tertiary hospital after the HST arrived. The written consent of HST transport should be obtained from parents before CCTT go out. After CCTT arrived at the local hospital and took over the children, an HST with the business-class should be notified immediately. The business-class carriage is the best choice for HST transport as it can provide enough space to accommodate the medical equipment, and for CCTT to perform some medical procedures during transport in case the patient’s condition is not stable. As HST cannot stop unless arriving at a station, so the CCTT cannot call for emergency help during transport. The CCTT should check all the equipment, medication, supplies before they leave the referring hospital. A complete and practical checklist is necessary for HST transport, which can improve CCTT-oriented workflow through both routine and unexpected situations for good outcomes^8^. During air medical transport, checklists can improve team performance through the prevention of errors and complications^9^. Meanwhile, it is essential for CCTT to make adequate communication with the crew of HST before initiation of transport. CCTT can refer to them for help during transport if necessary.

This study showed that HST transport was safe for critically-ill children. The vital signs after admission, including heart rate, respiratory rate, blood pressure and rectal temperature, showed no difference in both groups. The result of blood gas analysis and critical illness score at admission was almost no difference. Only the serum lactate in the ambulance group was higher than that in the HST group (Table 1). The underlying cause may be that the children in the ambulance group needed more time for medical transport; they could not receive further therapy timely in the tertiary center. The speed of HST ranges from 250 to 350 km/h in China, which can make medical transport more quickly over the ambulance. In China, the HST network is developing quite well, and many prefecture-level cities have high-speed railway stations (Fig. 4A)^6^. In addition, another evident advantage of HST transport is that CCTT can be rapidly delivered to the referring hospital by HST so that the critically-ill children could be under the management of CCTT as soon as possible. A recent study was against the “golden hour” in critically-ill children transport, continuing to emphasize the timely delivery of proper care over the speed of transfer^10^. Some studies have shown that specialized teams for transport could improve outcomes for proper treatment during transport^2,11,12^. Although sending a specialized team might be time-consuming and delay the critically-ill patients arriving at the tertiary care centers, robust evidence still shows that such teams should be worthy to consider for transport when available.

It is essential to highlight that this study confirmed that the mortality of children in the HST group showed no difference compared with the ambulance group. Proper care provided by CCTT for critically-ill children was critical to the consequence after transporting the children to the tertiary center^13^. In this study, the comprehensive life support on the HST ensured the stability of critically-ill children during transport. The children in HST could receive all necessary life support that is available in an ambulance, including oxygen therapy, intravenous infusion, invasive ventilation, and vital signs monitoring. The children in need of invasive ventilation were given routine ETCO_2_ monitoring during HST transport. Continuous ETCO_2_ monitoring followed by an arterial blood gas measurement of the PaCO_2_ would allow for a correlation of the PaCO_2_/ETCO_2_ concerning dead space ventilation. Some studies have shown that ETCO_2_ and PaCO_2_ are approximately correlated within 2 to 5 mmHg in healthy patients, but chronic disease states can cause this difference to increase, leading to ventilation errors^14,15^. The American Association of Respiratory Care clinical practice guidelines recommends ETCO_2_ as a method to guide ventilator management^16^. Our data also indicated that HST transport was more efficient and economical than the ambulance (¥ 2787.5 (1790, 4240) versus 2295(2208.75, 2380.5), *p* > 0.05, Table 2). Even longer transport distance and business class fare, there was still significantly less time-consuming, and less cost in the HST group. The critical point of pediatric critical transport is to deliver proper medical services and initialize life support for the children as soon as possible. Based on our data, HST transport has achieved this goal, which can offer quality specialty services more efficiently, and does not increase financial burden.

**Table 2:** The Primary and Secondary outcomes of ambulance and HST groups.

	Ambulance group	HST group	χ^2^/t	*P*
	(n = 80)	(n = 40)		
Mortality %(n)	12.5% (10)	15.0% (6)	0.144	0.704
Length of transport (h)	9.80 ± 5.68	6.39 ± 2.58	4.518	< 0.001*
Transport cost (Yuan, RMB)	2787.5 (1790, 3240)	2295 (2208.75, 3380.5)	0.293	0.770
Length of hospital stay(d)	29.76 ± 22.05	26.53 ± 18.60	0.797	0.427
Hospitalization cost (Yuan, RMB)	92,923.48 (54,715.65, 123,563.12)	89,756.71 (51,070.40, 115,645.76)	0.598	0.551

*p < 0.05.

In China, high-quality tertiary medical centers are mainly located in megacities, such as Beijing, Guangzhou and Shanghai^17^. These cities usually are the centers of HST network as well. HST transport network for critically-ill children could be established to cover most regions of this country. Without increasing financial burden, HST medical transport can be a potentially promising option to improve the outcomes of critically-ill children in developing countries or those countries with developed HST network.
